# Bioactive secondary metabolites with multiple activities from a fungal endophyte

**DOI:** 10.1111/1751-7915.12467

**Published:** 2016-12-19

**Authors:** Catherine W. Bogner, Ramsay S.T. Kamdem, Gisela Sichtermann, Christian Matthäus, Dirk Hölscher, Jürgen Popp, Peter Proksch, Florian M.W. Grundler, Alexander Schouten

**Affiliations:** ^1^ Institute of Crop Science and Resource Conservation (INRES)Department of Molecular PhytomedicineUniversity of BonnKarlrobert‐Kreiten Str. 1353115BonnGermany; ^2^ Institute of Pharmaceutical Biology and BiotechnologyHeinrich‐Heine‐University DüsseldorfUniversitäts Str. 1. Building. 26.2340225DüsseldorfGermany; ^3^ Institute of Photonic TechnologyWorkgroup Spectroscopy/ImagingAlbert‐Einstein‐Str. 907745JenaGermany; ^4^ Institute of Physical Chemistry and Abbe Center of PhotonicsFriedrich Schiller UniversityHelmholtzweg 407743JenaGermany; ^5^ Research Group Biosynthesis/NMRMax Planck Institute for Chemical EcologyHans‐Knöll‐Str. 807745JenaGermany; ^6^ Present address: Organic Plant Production and Agroecosystems Research in the Tropics and Subtropics (OPATS)University of KasselSteinstr. 1937213WitzenhausenGermany; ^7^ Present address: Laboratory of NematologyWageningen UniversityDroevendaalsesteeg 16708 PDWageningenThe Netherlands

## Abstract

In order to replace particularly biohazardous nematocides, there is a strong drive to finding natural product‐based alternatives with the aim of containing nematode pests in agriculture. The metabolites produced by the fungal endophyte *Fusarium oxysporum* 162 when cultivated on rice media were isolated and their structures elucidated. Eleven compounds were obtained, of which six were isolated from a *Fusarium* spp. for the first time. The three most potent nematode‐antagonistic compounds, 4‐hydroxybenzoic acid, indole‐3‐acetic acid (IAA) and gibepyrone D had LC_50_ values of 104, 117 and 134 μg ml^−1^, respectively, after 72 h. IAA is a well‐known phytohormone that plays a role in triggering plant resistance, thus suggesting a dual activity, either directly, by killing or compromising nematodes, or indirectly, by inducing defence mechanisms against pathogens (nematodes) in plants. Such compounds may serve as important leads in the development of novel, environmental friendly, nematocides.

## Introduction

Plant‐parasitic nematodes pose a problem in agriculture by significantly affecting plant growth and crop yield at a global scale (Jones *et al*., 2013). The availability of resistant plant varieties is limited (Onkendi *et al*., 2014), and the most effective nematocides are unfortunately also the most hazardous from an environmental and human health perspective (Fuller *et al*., 2008). Evidently, there is currently strong pressure in driving these toxic nematocides from the market, leaving the grower with only moderately effective chemicals, which generally have nematostatic rather than nematocidal activity. Unless alternative methods or chemicals to contain nematode proliferation in the field become available, crop losses caused by nematodes may be further aggravated in future.

A potential opportunity to control nematode damages in crops is the use of endophytes and their secondary metabolites. Endophytes are generally defined as facultative plant‐colonizing microorganisms that do not cause disease symptoms in the plant (Hyde and Soytong, 2008). Their ability to provide quantitative resistance towards nematodes is still not well understood. There is evidence that endophytes may affect nematodes either directly, by synthesizing nematocidal compounds that kill or paralyse nematodes, or indirectly by triggering plant defence responses that are aimed at the nematode (Schouten, 2016). This knowledge gap is obstructing further development of endophytes or their metabolites towards an effective means of controlling nematodes in the field.

One of the endophytes that have been intensively studied with respect to nematode control is Fo162. This is a strain of the *Fusarium oxysporum* species complex (FOSC) that has been shown to reduce nematode infection, development and fecundity (Martinuz *et al*., 2013). This effect is mostly attributed to systemic induced resistance mechanisms inside the plant (Martinuz *et al*., 2012), although it was also demonstrated that Fo162 was capable of producing nematocidal compounds (Hallmann and Sikora, 1996). However, the responsible metabolites were never identified. In this study, we fully characterized a number of compounds that can be synthesized by Fo162, some of which do have nematocidal activity against the economically important root‐knot nematode, *Meloidogyne incognita* (Hu *et al*., 2013).

Remarkably, one of the best performing nematocidal compounds is in fact a known phytohormone, indicating the multiple roles that natural products from endophytes can play in defence against nematodes. This finding forces us to reconsider the role of particular compounds in host–pathogen interactions and further emphasizes that endophytes can serve as a valuable reservoir for finding effective natural compounds with both a direct and an indirect activity towards nematodes.

## Results

### Identification of compounds from Fo162

**Figure 1:**
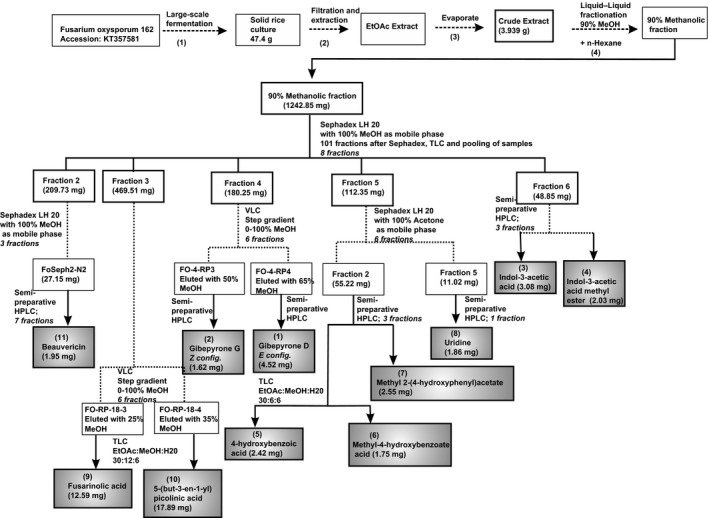
Flow chart illustrating the process of extraction and fractionation of bioactive compounds produced by endophytic *Fusarium oxysporum* 162 on solid rice media. All compounds (1‐11) are highlighted in grey. Numbers in parentheses are dry weights (mg) of fractions.

**Figure 2:**
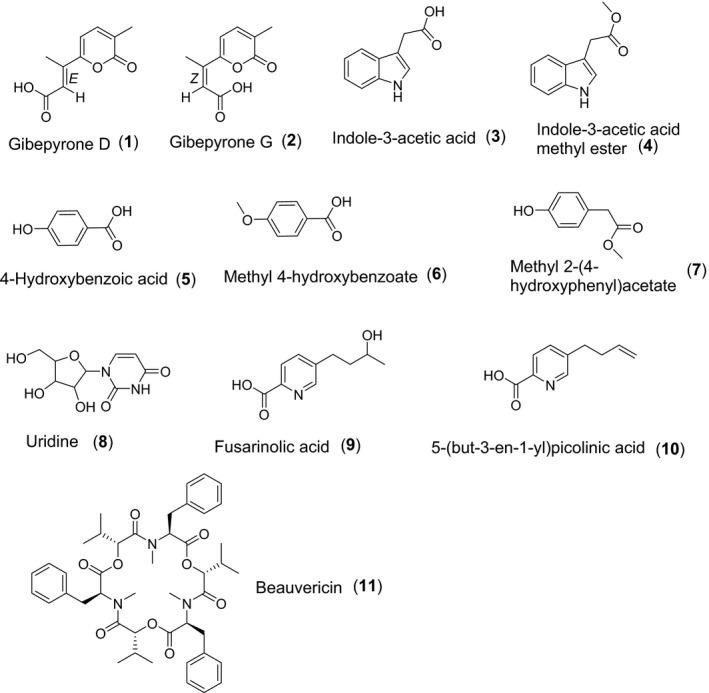
Structures of compounds **1‐11** isolated from endophytic *Fusarium oxysporum* 162, grown on solid rice media.

Fungal metabolites have primarily served as lead structures for the development of nematocidal compounds, but so far only few reports have mentioned such compounds isolated from *F. oxysporum*. The secondary metabolites produced in rice media by Fo162 [originally isolated from the cortical tissue of surface‐sterilized tomato roots cv. Moneymaker in Kenya by Hallmann and Sikora (1994)] were studied and eleven known compounds were isolated. The compounds were isolated according to various procedures as illustrated in Fig. 1. The chemical structures of the compounds are shown in Fig. 2. A summary of the detailed NMR descriptions of all the pure compounds is given in Figs S1–S10. Literature comparison of all compounds was in agreement with the obtained NMR data. The compounds were identified as: gibepyrone D (**1**), gibepyrone G (**2**), indole‐3‐acetic acid (**3**), indole‐3‐acetic acid methyl ester (**4**), 4‐hydroxybenzoic acid (**5**), methyl 4‐hydroxybenzoate (**6**), methyl 2‐(4‐hydroxyphenyl)acetate (**7**), uridine (**8**), fusarinolic acid (**9**), 5‐(but‐3‐en‐1‐yl)picolinic acid (**10**) and beauvericin (**11**). Our study of Fo162 metabolites led to the isolation of at least seven bioactive compounds, six of which were purified from this fungal species for the first time. These were compounds **3**,** 4**,** 5**,** 6**,** 7** and **8**.

### Nematocidal activities of isolated metabolites against *M. incognita*

The nematocidal activity of compounds **1‐11** were examined in *in vitro* bioassays on *M. incognita* J2 larvae. The frequently used carbamate‐ and organophosphate‐based commercial granular nematicides Furadan^®^ and Temik^®^ 10G contain carbofuran and aldicarb, respectively. Carbofuran and aldicarb were therefore used as positive controls. In the first series of bioassays, the degree of mortality after 24, 48 and 72 h of each compound at the highest concentration of 400 μg ml^−1^ was assessed and the compounds were divided into five categories, based on the mortality rates achieved by each compound. These categories were as follows: no effect (0% death), poor (0–25% death), moderate (26–50% death), good (51–75% death) and strong (71–100% death) (Table S1). Three compounds namely 4‐hydroxybenzoic acid (**5**), indole‐3‐acetic acid (**3**) and gibepyrone D (**1**) had strong mortality activity. Preliminary assays (Table S1) indicated that at the highest tested concentration (400 μg ml^−1^), nearly 100% of *M. incognita* J2 larvae died after 72 h of contact with compounds **5**,** 3** and **1**. Similar results were observed for positive control 1 (carbofuran), while positive control 2 (aldicarb) had a much weaker lethal activity (44%).

The negative control (1% methanol) was tolerated by the *M. incognita* J2 larvae and did not lead to significant nematode death. The next most effective compound was methyl 2‐(4‐hydroxyphenyl)acetate (**7**), with a good mortality rate of 58% followed by three compounds namely indole‐3‐acetic acid methyl ester, methyl 4‐hydroxybenzoate and gibepyrone G (**4, 6** and **2**), which elicited moderate mortality rates of 45, 38 and 37% respectively. Fusarinolic (**9**) and picolinic acid (**10**) had poor mortality rates against *M. incognita* while uridine (**8**) and beauvericin (**11**) were not effective. To ensure that a nematocidal and not a nematostatic effect was observed, nematodes were transferred to water for 24 h after exposure to the compounds, and their mobility assessed again. The nematodes that remained immobile were considered dead.

**Figure 3:**
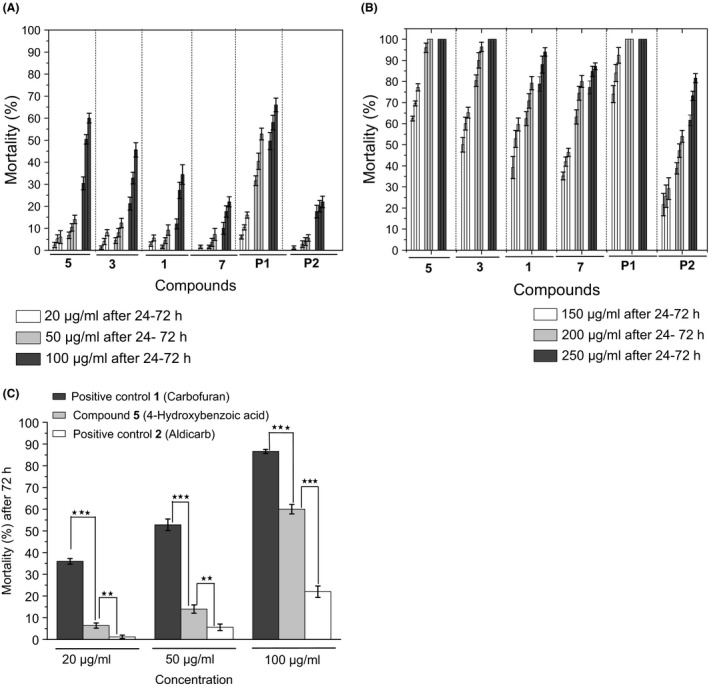
Mortality rates of *M. incognita* J2 larvae after 24, 48 and 72 h contact with four Fo162 metabolites: 4‐hydroxybenzoic acid (**5**), indole‐3‐acetic acid (**3**), gibepyrone D (**1**) and methyl 2‐(4‐hydroxyphenyl)acetate (**7**) and the nematicides carbofuran (**P1**) and aldicarb (**P2**) as positive controls. (A) Dose‐dependent mortality test in 20, 50 and 100 μg ml^−1^. (B) Dose‐dependent mortality test in 150, 200 and 250 μg ml^−1^. (C) Comparison between the most potent compound **5** and the two positive controls. A compound was considered lethal when it caused significantly (*P *≤* *0.05) high percentage of nematode death compared with the negative control (1% methanol). Data are expressed as the means ± standard errors of five replicates. Significance was tested according to Holm–Sidak multiple comparisons versus control group using Sigma plot 12.5. Means followed by asterisks (*** = *P < *0.001; ** = *P < *0.01) are significantly different from the mean percentage of dead nematodes in the negative control.

In the second series of bioassays, the nematodes were subjected to six different concentrations of the compounds (20, 50, 100, 150, 200 and 250 μg ml^−1^) to assess the dose necessary for nematodes to be killed. Mortality rates of *M. incognita* J2 larvae after 24, 48 and 72 h contact with four Fo162 metabolites: 4‐hydroxybenzoic acid (**5**), indole‐3‐acetic acid (**3**), gibepyrone D (**1**) and methyl 2‐(4‐hydroxyphenyl)acetate (**7**) and the nematicides carbofuran (**P1**) and aldicarb (**P2**) as positive controls were evaluated. These results are depicted in Fig. 3 (A–C). A compound was considered lethal when it caused significantly (*P *≤* *0.05) high percentage of nematode death compared with the negative control (1% methanol). Here, a concentration‐dependent effect of the compounds was observed. At incubation times from 24 to 72 h, the percentage of dead nematodes versus the total number of nematodes increased for the majority of the compounds from non‐significant at an initial concentration of 20 μg ml^−1^ to significant levels at higher concentrations. In comparison with the positive controls (Fig. 3C), significant differences could already be observed at a concentration of 20 μg ml^−1^.

The dose–response testing allowed the calculation of the LC_50_ values of the compounds after 24, 48 and 72 h (Table 1). Three of the eleven tested compounds produced high mortality rates, as in the preliminary screening. 4‐Hydroxybenzoic acid (**5**) was again the most potent compound, with LC_50_ values of 129, 115 and 104 μg ml^−1^ after 24, 48 and 72 h of treatment respectively. As in the preliminary assay, the next most effective compounds were indole‐3‐acetic acid (**3**) and gibepyrone D (**1**). Nematocidal activity increased when the exposure time increased to 72 h. The LC_50_ 72 h values were 117 and 134 μg ml^−1^, respectively, for **3** and **1**. The LC_50_ 72 h values for the positive controls carbofuran and aldicarb were 64 and 180 μg ml^−1^ respectively. These results revealed that carbofuran had a higher activity (almost twofold) than the most potent compound (**5**) isolated from Fo162. However, the activity of 4‐hydroxybenzoic acid (**5**) was stronger than that of aldicarb. The four best performing compounds isolated from Fo162 (**5**,** 3, 1** and **7)** were purchased commercially and tested in subsequent bioassays, yielding results similar to those of the isolated compounds.

**Table 1:** LC_50_ and *R* ^2^ values of potential nematocidal metabolites against *M. incognita* at 24, 48 and 72 h after treatment

Number	Compound	LC_50_ (μg ml^−1^)		LC_50_ (μg ml^−1^)		LC_50_ (μg ml^−1^)	
		24 h	*R* ^2^	48 h	*R* ^2^	72 h	*R* ^2^
**1**	Gibepyrone D: *E* configuration	175.26	0.94	149.50	0.98	134.31	0.98
**2**	Gibepyrone G: *Z* configuration	365.58	0.80	309.20	0.84	265.57	0.87
**3**	Indole‐3‐acetic acid	141.13	0.96	127.97	0.98	117.28	0.98
**4**	Indole‐3‐acetic acid methyl ester	303.67	0.93	255.07	0.94	218.57	0.96
**5**	4‐Hydroxybenzoic acid	129.03	0.96	115.46	0.95	104.84	0.93
**6**	Methyl 4‐hydroxybenzoate	356.61	0.88	296.07	0.91	253.24	0.94
**7**	Methyl 2‐(4‐hydroxyphenyl)acetate	180.50	0.93	158.58	0.95	149.22	0.96
**8**	Uridine	–	–	–	–	–	–
**9**	Fusarinolic acid	651.48	0.91	624.20	0.88	600.79	0.92
**10**	5‐(But‐3‐en‐1‐yl)picolinic acid	705.11	0.94	679.47	0.90	655.23	0.87
**11**	Beauvericin	–	–	–	–	–	–
Positive Control 1	Carbofuran	102.96	0.95	95.50	0.93	64.15	0.80
Positive Control 1	Aldicarb	234.88	0.94	200.68	0.94	180.78	0.94

### Raman microspectroscopy

**Figure 4:**
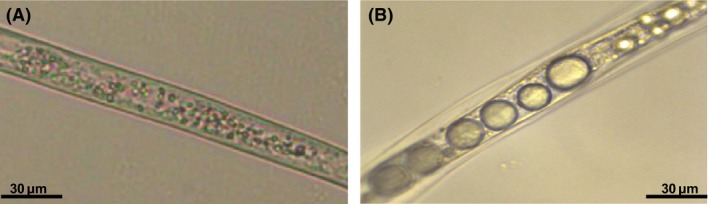
Bright field microscopic images of *M. incognita* morphology from bioassay treatment with 4‐hydroxybenzoic acid (**5**). Untreated nematode and treated nematode are indicated in figures A and B respectively. Visible vacuole‐like droplets can be seen inside the body (middle region) of the nematode (B).

**Figure 5:**
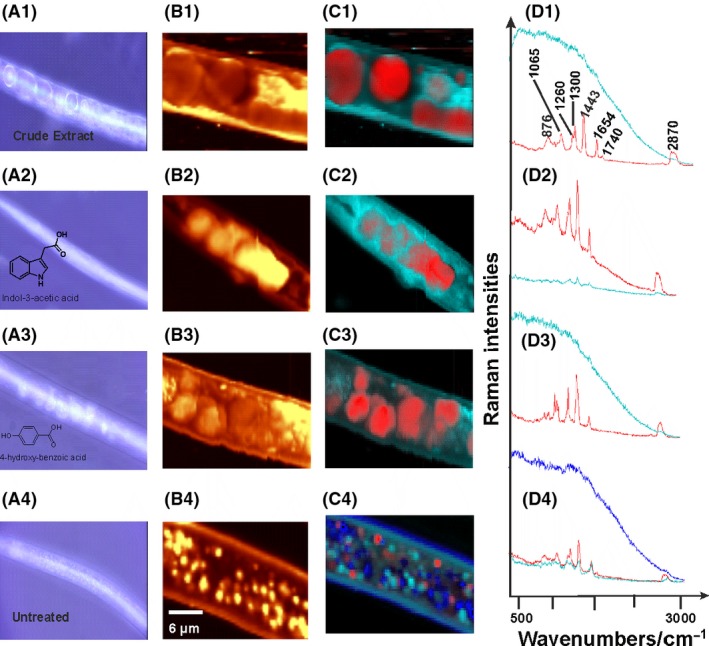
Raman spectra of nematodes treated with compounds. Figures A1–A4 show bright field (BF) images of *M. incognita* treated with crude extract, compound (**3)** indole‐3‐acetic acid, compound (**5**) 4‐hydroxybenzoic acid and untreated respectively. Figures B1–B4 are the Raman images of the BF images. Images were generated by integrating the intensities of the C–H stretching vibrations which are characteristic for organic molecules. Figures C1–C4 were reconstructed from figures B1–B4 using spectral decomposition algorithm as described in the in‐house literature (methods section) while Figures D1–D4 represent the associated Raman spectra information.

As early as 48–72 h after treating the nematodes with the active compounds, vacuole‐like structures were observed in the middle and tail parts of the nematode body, although no droplets were observed in the head region of the nematode. Figure 4 shows bright field images of an untreated and treated nematode, with the latter showing the vacuole‐like structures. Compounds that resulted in poor or no nematode mortality did not lead to the formation of these droplets. The chemical composition of the vacuole‐like droplets was characterized using confocal Raman spectroscopy (Fig. 5 A–D). At a glance, the spectrum exibited typical features for lipids (depicted in red; Fig. 5: D1–D4).

The lipid profile is dominated by unsaturated fatty acids due to the presence of marker peaks centred at 1655/cm which represent the stretching of C=C of unsaturated side‐chains and a weak bond located at 1740/cm, which corresponds to the C=O stretching of the ester bond. The distinctive aliphatic intensities of C‐H stretching bands between 2800 and 3100 cm^−1^ and C‐H deformation bands near 1300 and 1440 cm^−1^ were more intense in the red curves, whereas the signals resulting from proteins (light blue curves) were too weak and appeared mostly as fluorescence (no peaks). Further bands are assigned to C‐C groups at 1070 cm^−1^. Reference spectra and detailed assignments of Raman spectra of biological molecules are described in the literature (de Gelder *et al*., 2007). A slight difference was observed in the untreated sample (Fig. 5: D4). The fluorescence background from the protein regions (light and dark blue spectra) was partially more pronounced in comparison with the treated samples. Due to the fact that most of the protein regions were dominated by fluorescence in treated and untreated samples, their Raman spectra appeared only as weak signals, and therefore, they were not described further in this experiment.

**Figure 6:**
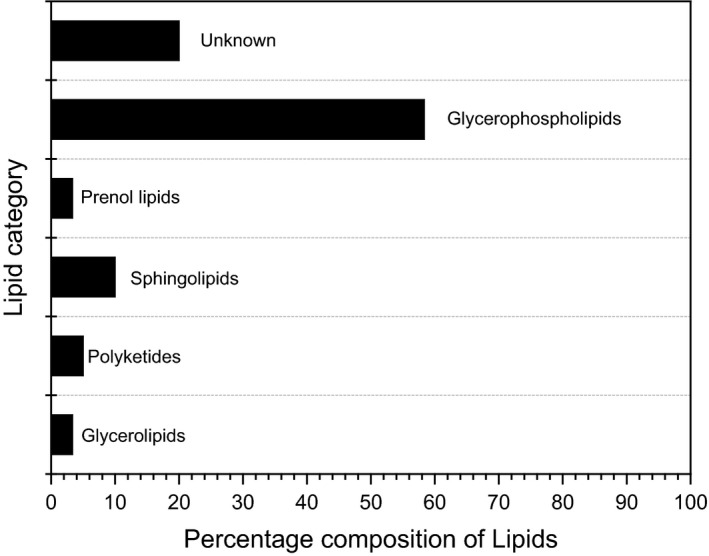
Relative composition of detected lipids in *M. incognita* after treatment with the crude extract.

The lipids identified by MS analysis are shown in Fig. 6 and the Table S2. The most abundant lipid component was glycerophospholipids (58.33%). Other lipids included sphingolipids (10%), polyketides (5%), prenol lipids (3.33%) and glycerolipids (3.33%). Twenty percent of the lipid composition was unknown as no results could be obtained from the lipid gateway website. Most glycerophospholipids were observed in the *m/z* range of 700–900 while majority of the unknown lipids were found in the *m/z* range of 900–1000. Important to note is that there were no obvious lipid signal peaks detected in the *m/z* range of 1000–1300.

## Discussion

**Figure 7:**
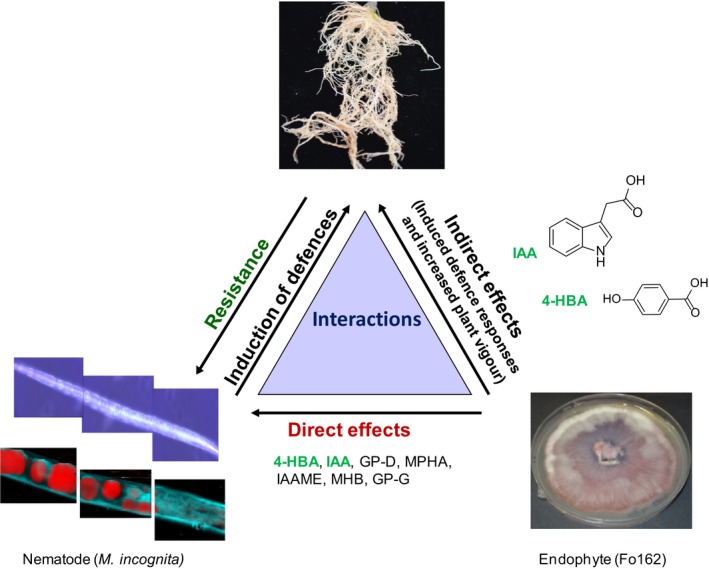
Diagram showing the tripartite interactions among plant, endophyte and nematode. The endophyte (Fo162) can induce nematode resistance through direct and indirect mechanisms. In the plant, defence responses towards the nematode and vigour can be increased by the endophyte‐produced 4‐hydroxybenzoic acid (4‐HBA) and indole‐3‐acetic (IAA). Additionally, the phytohormone (IAA) and the salicylic acid isomer (4‐HBA) have a dual function and can, together with other metabolites produced by the endophyte, be involved in the killing of the nematode, *M. incognita*. GP‐D, gibepyrone D; MPHA, methyl 2‐(4‐hydroxyphenyl)acetate; IAAME, indole‐3‐acetic acid methyl ester; MHB, methyl 4‐hydroxybenzoate; GP‐G, gibepyrone G.

The ability of non‐pathogenic endophytic *F. oxysporum* strain Fo162 to successfully reduce nematode penetration, subsequent galling as well as reproduction in inoculated tomato plants led us to isolate and determine the antagonistic activities of secondary metabolites produced by this strain against the root‐knot nematode *M. incognita*. Fo162 is capable of producing several different active compounds, some of which have a potential dual activity, affecting not only the nematode but also plant physiology. A diagram illustrating this tripartite interaction and the dual activity of two constituents is shown in Fig. 7. Four of the characterized compounds, gibepyrone D (E configuration) (**1**) indole‐3‐acetic acid (IAA) (**3**), 4‐hydroxybenzoic acid (4‐HBA) (**5**) and methyl 2‐(4‐hydroxyphenyl)acetate (**7**), have nematocidal activities that fall between the included commercial and frequently applied reference compounds, carbofuran and aldicarb (Table 1). One of the best performing compounds from the bioassays was IAA (**3**) (LC_50_ 72 h: 117 μg ml^−1^), a well‐known phytohormone. Like other auxins, the effect of IAA in plants is versatile and concentration dependent, generally stimulating growth, like providing apical dominance in the shoot, stimulating shoot growth, fruit development and the formation of lateral roots (Ivanchenko *et al*., 2008). Between auxin and the jasmonate/ethylene (JA/ET) defence pathways, a hormonal cross‐talk in the primary root was observed (Ortega‐Martinez *et al*., 2007; Pieterse *et al*., 2012) and, more recently, it was also shown that auxins positively affected stress tolerance of the plant (Kerchev *et al*., 2015). In addition to plants, many soil‐borne bacteria and fungi are capable of producing IAA (Duca *et al*., 2014) and there is evidence that such microorganisms can change root architecture (Zamioudis *et al*., 2013). Our endophyte, Fo162, stimulated development and lateral root formation of the model plant *Arabidopsis thaliana* (Martinuz *et al*., 2015). Until now, no indole alkaloids have been reported with nematocidal effects and IAA toxicity towards nematodes adds a completely new dimension to the role of this compound in plant defence. By generating IAA, Fo162 can simultaneously increase tolerance to stress (nematodes) in plants and directly kill nematodes.

Remarkably, the most potent nematocidal compound, 4‐HBA (**5**) (LC_50_ 72 h: 104 μg ml^−1^), also has the capability of increasing stress tolerance. Exogenously added 4‐HBA improved the drought tolerance of winter wheat and the freezing tolerance of spring wheat, whereas its structural analogue, salicylic acid, reduced the freezing tolerance of winter wheat and the drought tolerance of spring wheat (Horváth *et al*., 2007). Aoudia *et al*. (2012) tested the effect of phenolic compounds, two of those being 4‐hydroxybenzoic acid and salicylic acid, for their paralysing effect on *M. incognita*. These compounds were demonstrated to paralyse nematodes (EC_50_ at 24 h) at a concentration of 871 μg ml^−1^ for 4‐HBA and 379 μg ml^−1^ for salicylic acid (Aoudia *et al*., 2012). However, their assay set‐up seemed to have been rather crude and incomplete as much lower toxicity concentrations for salicylic acid (MIC_50_ 24 h: 61 μg ml^−1^) on *M. incognita* were reported (Wuyts *et al*., 2006). Another study by Nguyen *et al*. (2013) revealed that a compound related to 4‐HBA, namely 3, 4‐dihydroxybenzoic acid, caused 47.5% mortality among *M. incognita* after 12 h at a concentration of 250 μg ml^−1^ (Nguyen *et al*., 2013). The LC_50_ values in their study were not determined. Comparison of the nematocidal activities of 4‐HBA (**5**) and those of methyl‐4‐hydroxybenzoate (**6**) in our work indicated that the latter was less active towards *M. incognita*. The site(s) and number of hydroxyl groups on the phenol group are thought to have a correlation with their toxicity to microorganisms, with evidence that increased hydroxylation results in increased toxicity and that methoxy groups seem to abolish activity. However, this aspect on structure–activity relationship is contradictory because other findings argue that fewer hydroxyl groups are more lipophilic and thus more membrane disruptive (Cowan, 1999). In our work, we could not draw universal structure–activity relationship conclusions that are consistent for all compounds.

Another well‐performing nematocidal compound, produced by Fo162, was gibepyrone D (**1**) (*E* configuration: LC_50_ 72 h: 134 μg ml^−1^). Simple pyrones, belonging to the class of monocyclic α‐pyrones, were reported to have remarkable biological effects ranging from antifungal, antibacterial, antitumor activities as well as use as pheromones and yeast biocontrol agents (Schäberle, 2016). Experimental evidence also demonstrated that 6‐pentyl‐α‐pyrone isolated from *Trichoderma koningii* reduced rhizoctonia root rot of wheat (Worasatit *et al*., 1994). There is currently only one report showing the nematocidal effect of aspyrone D (*E* configuration) isolated from *Aspergillus melleus* against the root‐lesion nematode *Pratylenchus penetrans*. There, 39% of the tested nematodes died at a concentration of 100 μg ml^−1^ after 48 h (Kimura *et al*., 1996). In our work, we were also able to isolate the isomer of gibepyrone D which contains the *Z* configuration and was named gibepyrone G (**2**). There was a significant difference in nematocidal activity between these two α‐pyrones because the *E* isomer had an almost twofold higher activity than the *Z* isomer. Due to the fact that the occurrence of the *Z* isomer is very rare in nature (Abraham and Arfmann, 1988), we hypothesized that the readily available form of the natural product (*E* isomer) is likely to be biologically more active than its *Z* isomer. Furthermore, a natural selection for toxic compounds may be advantageous, as microorganisms are in constant competition with one another. This is the first report of the nematocidal activity of α‐pyrone *Z* isomer. The effect of gibepyrone D in plant development, when at all, is currently unknown.

Another isolated compound in our research was methyl 2‐(4‐hydroxyphenyl)acetate (**7**) which had a good nematocidal activity (LC_50_ 72 h: 149 μg ml^−1^). This compound has previously been isolated from an endophytic bacterial strain, *Nocardia* sp. (Li *et al*., 2015) and the fungal endophytes *Penicillium chrysogenum* (Peng *et al*., 2011) and *Trichoderma polysporum* (Kamo *et al*., 2016). Li *et al*., 2015 tested for antibacterial activity against *E. coli* and *Staphylococcus aureus*, antifungal activity against *Candida albicans* and antioxidant activity and, in all cases, no obvious activity was detected. Its nematocidal activity has never been reported before.

The compound beauvericin (**11**) had no effect against *M. incognita*. Weak activities of beauvericin against *M. incognita* (Li and Zhang, 2014) and the free‐living bacterial feeding nematode *Caenorhabditis elegans* were reported (Shimada *et al*., 2010). The same compound, however, showed activity against the pine wood nematode *Bursaphelenchus xylophilus* (Shimada *et al*., 2010). Finally, fusaric acid analogues, namely fusarinolic acid (**9**) and picolinic acid (**10**), showed poor nematocidal activity.

In our assays, only the toxicity of the individual compounds was determined. Kwon *et al*. (2007) provided evidence that when two metabolites were applied together in a ratio of 1:1, the mixture showed more potent activity. It may thus be that the compound mixture produced by the endophyte is much more potent towards nematodes, in which the individual poorly performing compounds still play an important additive role. From our own findings and literature results, it is evident that future results about testing the general nematicidal pattern in other nematode species would be very informative in further evaluating the potency of the compounds. The fact that IAA and 4‐HBA can readily be obtained commercially at significant amounts facilitates such screening.

We also investigated the phenotype caused by the active compounds on nematodes. The positive control, carbofuran, displayed the same vacuole‐like morphology inside the nematode body as that found with the Fo162 nematocidal compounds while aldicarb did not show such phenotype. We therefore characterized the composition of the vacuole‐like droplets using Raman spectroscopy and confirmed that the observed droplets contained lipids. The second‐stage juvenile (J2 larvae) of the root‐knot nematode *M. incognita* is an obligate biotroph that depends on its host for survival. For a successful parasitic lifestyle, different behavioural strategies have been suggested. These mainly include the consumption of its lipid reserves during starvation or prior to finding a host (Spiegel and McClure, 1995).

Hölscher *et al*. (2015) observed that once the banana nematode *Radopholus similis* was treated with the nematocidal compound anigorufone, bulky oil droplets containing anigorufone inside the nematode body were formed. On the basis of similar observations as supported by our data, we believe that lipid formation may be one strategy the nematode employs to overcome or minimize the toxic effects of the secondary metabolites. Additionally, we hypothesize that formation and accumulation of lipid droplets is a marker for death because the metabolism of the nematode has been affected in a negative way.

Genome analyses performed on *M. incognita* J2 larvae found that many genes and key pathways were similar to *C. elegans* L2 (dauer larvae), thereby providing experimental support that the J2 larval stage could be viewed as a functional equivalent of *C. elegans* dauer larvae (McCarter *et al*., 2003). For *C. elegans*, it was demonstrated that alterations in lipid metabolism and intestinal oil droplets were involved in minimizing the toxicity of polychlorinated biphenyl (Menzel *et al*., 2007). Another remarkable finding in our work was that the most abundant lipids after treating the nematodes with secondary metabolites crude extract were glycerophospholipids. Glycerophospholipids are major components of cellular membranes and they play important roles in various cellular functions including signal transduction, vesicle trafficking and membrane fluidity (Hishikawa *et al*., 2014). Carbofuran, too, did show the same phenotype as the Fo162 toxic compounds, whereas aldicarb did not. It was previously shown that *C. elegans* hermaphrodites responded to the toxic ∂‐endotoxin Cry5B by, among other phenotypes, the formation of unidentified vacuole‐like structures in gut cells and a withering of the gut (Marroquin *et al*., 2000), similar to what we observed with our isolated toxic compounds in *M. incognita* J2 larvae. However, our *M. incognita* J2 larvae were still in the preparasitic phase and do not feed yet, which differs from the *C. elegans* hermaphrodite. Unfortunately, we were not able to successfully analyse the uptake of our toxic compounds inside the nematodes body because the lipid droplets fused together, making it difficult to perform MS imaging. We therefore assumed that these compounds may have been taken up in the nematode body. We believe that the possibility of these compounds forming complexes with nematodes putative lipid molecules could be of interest for future studies. Furthermore, we think that a deeper understanding of this area in plant‐parasitic nematodes will reduce model hopping between *C. elegans* and plant‐parasitic nematodes.

Although the genus is notorious for its plant pathogenic species, which can produce an array of toxins, *Fusarium* species or particular isolates of these species have also been found to reside as endophytes inside plants without causing disease symptoms. Several investigations have proven that fungi of the genus *Fusarium* are a rich source of biologically active secondary metabolites including antibacterial and antifungal agents, fungal toxins and immunosuppressive compounds (Wang *et al*., 2011). However, studies with regard to the nematocidal activities of natural compounds from the species *Fusarium oxysporum* have been rarely reported and, if at all mentioned, they mostly relied on the nematocidal effects of culture filtrates without detailed studies on the identification of toxic metabolites (Hallmann and Sikora, 1996). Up to now, only two nematocidal compounds, bikaverin and fusaric acid, were obtained by bioassay‐directed fractionation from the fungus *Fusarium oxysporum*. Their nematocidal activities were tested against pine wood nematode *Bursaphelenchus xylophilus* (Kwon *et al*., 2007).

The best performing commercial nematicide in our assay, carbofuran, has already been blacklisted and its import and export into the European market have been minimized (office, P. Commission Regulation 2011). Carbofuran poses various risks to human health, e.g., headache, chest pain, nausea, diarrhoea, permanent damage to the nervous system and the reproductive system. It is also responsible for the poisoning of domestic and wild animals (Ruiz‐Suárez *et al*., 2015). In view of this, there is an urgent need to replace such compounds with other more environmentally sound alternatives. Our study has led to the identification of several bioactive compounds produced by *F. oxysporum* Fo162, some of which can exert a dual activity. These latter compounds in particular may be highly relevant for the development into future commercial nematocides, as the dual activity may complicate the development of resistance within the nematode population, thus making such nematocides more effective and lasting. By formulating and combining these bioactive compounds, which may even be modified, the overall nematocidal activity may be increased and by means of field trials the efficacy, the longevity of these compounds in different soil types and their effects on crops and crop yield will have to be determined. And, as already mentioned, the activity of the characterized compounds towards other plant‐parasitic nematode species still has to be assessed. Nevertheless, *M. incognita* is considered the most widespread and damaging plant‐parasitic nematodes in the tropics and subtropics, having an extremely wide host range, like tomato, soya bean, cassava and banana (Trudgill, 1997; Luc *et al*., 2005), and an effective control of this species alone would already be most valuable.

## Experimental procedures

### Chemical and materials

Solvents were purchased in analytical grade from Fisher Scientific (Schwerte, Germany) or Merck (Darmstadt, Germany). NMR solvents were obtained from Euriso‐top GmbH (Saarbrücken, Germany). Nematode bioassay chemicals were purchased from Sigma‐Aldrich (Steinheim, Germany) or Carl‐Roth (Karlsruhe, Germany).

### General experimental procedures

For column chromatography, Merck MN Silica gel 60M (0.04–0.063 mm) or Sephadex LH20 were used as stationary phases. Thin‐layer chromatography (TLC) was performed using pre‐coated silica gel 60 F254 TLC plates (Merck). Detection of the compounds on the TLC plates was obtained by observing the absorption at 254 and 366 nm under a UV lamp or the compounds were visualized by spraying with anisaldehyde reagent. Analytical HPLC was carried out on a Dionex UltiMate 3400 SD with a LPG‐3400SD Pump coupled to a photodiode array detector (DAD3000RS); routine detection was carried out at 235, 254, 280 and 340 nm. The separation column (5 μm; 125 × 4 mm) was prefilled with Eurospher 100 C18 (Knauer, Berlin, Germany). Methanol and 0.1% formic acid in H_2_O were used as the mobile phase with 1.0 ml min^−1^ flow rate. The following gradient was applied as a regular program: 0 min (10% MeOH), 5 min (10% MeOH), 35 min (100% MeOH) and 45 min (100% MeOH).

Semi‐preparative RP‐HPLC was conducted using a HPLC Merck Hitachi system (Pump L7100 and UV detector L7400) and a Eurospher 100–10 C18 (Knauer) column (10 μm; 300 × 8 mm). Methanol and 0.1% trifluoroacetate in water were used as the mobile phase with a flow rate of 5.0 ml min^−1^. The mobile system program and the UV wavelength for the target compounds were set according to the retention time from the analytic HPLC. Target peaks were collected manually during the running of the program.

Mass spectra data were collected on a LC‐MS HP1100 Agilent Finnigan LCQ‐Deca mass spectrometer (Thermo Finnigan,Bremen, Germany) while high‐resolution mass (HRESIMS) spectra were recorded with a FTHRMS‐Orbitrap (Thermo Finnigan, Bremen, Germany) mass spectrometer. Chemical structures of the isolated compounds were determined by one‐ and two‐dimensional nuclear magnetic resonance (NMR) spectroscopy. ^1^H, ^13^C and 2D NMR spectra were recorded at 25°C in deuterated methanol‐*d*_4_ or DMSO‐*d*_6_ on Bruker ARX 300 or AVANCE DMX 600 NMR spectrometers. Chemical shifts are reported in ppm (δ), using CD_3_OD as the solvent (unless otherwise stated) and tetramethylsilane (TMS) as the internal standard.

Multiplicities are described using the following abbreviations: s = singlet, d = doublet, t = triplet, q = quartet, m = multiplet. ^1^H and ^13^C NMR assignments were supported by ^1^H−^1^H correlation spectroscopy (COSY), heteronuclear multiple‐bond correlation (HMBC) or heteronuclear single‐quantum correlation spectroscopy (HSQC) experiments.

### Fungal material and cultivation

The non‐pathogenic fungal endophyte Fo162 that was isolated from healthy tomato roots cv. Moneymaker was identified during our previous study (Bogner *et al*., 2016) based on a multigene DNA sequence analysis. The GenBank Accession numbers of ITS, ß‐tubulin and TEF1α gene regions are KT357581, KT316682 and KT357523 respectively. A voucher strain has been deposited in the culture collection of Molecular Phytomedicine, University of Bonn, Germany, with the ID number Fo162. Large‐scale fermentation was carried out in ten Erlenmeyer flasks (1 l each) on solid rice medium (Milch‐Reis, ORYZA^®^). Distilled water (100 ml) was added to 100 g commercially available rice and autoclaved. The autoclaved rice medium in each Erlenmeyer flask was inoculated by adding five plugs of an 8 day old culture of Fo162 grown on PDA. The flasks were then incubated at room temperature (22°C) under static conditions for 28 days.

### Extraction and isolation of fungal cultures

The rice culture was chopped into small pieces with a sterile spatula and 250 ml of EtOAc was added to each flask. Each fermented rice substrate was extracted three times with EtOAc (3 × 250 ml) on a shaker (Certomat R/SII; Sartorius, Göttingen, Germany) for 45 min at room temperature (22°C). The fungal material (47.4 g) was removed by filtration through a Whatman filter paper. The extracts were combined and concentrated to dryness by rotary evaporation at 40°C (Rotavapor R‐215; Buchi, Flawil, Switzerland) under vacuum to yield crude extracts (3.93 g). The obtained crude extract was analysed by HPLC. Initial purification was achieved by successively partitioning the crude extract between *n*‐hexane and 90% aqueous MeOH. A preliminary assay of the two layers (*n*‐hexane and 90% MeOH) against *M. incognita* was performed, and the 90% methanolic extract showed the strongest activity. Further compound isolation was carried out using the methanolic extract. Evaporation of the 90% MeOH fraction yielded 1242.85 mg. Briefly, the 90% methanolic fraction was further separated by Sephadex LH 20 column chromatography using MeOH as the mobile phase to yield 101 fractions. These fractions were further analysed by TLC, and the solvent system used was EtOAc, MeOH and water in a ratio of 30:5:4. Visualization was carried out by spraying the plates with anisaldehyde reagent. Fractions that had similar compositions, as indicated by colour and location of the TLC spots, were pooled to yield a total of eight fractions. Separation was carried out using size exclusion chromatography over Sephadex LH 20. Vacuum liquid chromatography (VLC) on silica gel 60 using MeOH step gradient elution was also employed for separation. Additionally, preparative TLC was used for isolation. Bands of target compounds were marked under UV light and cut out. The compounds were then eluted with MeOH. Other fractions were purified by semi‐preparative HPLC. The obtained pure fractions were numbered **1‐11**. The amount obtained for the compounds was as follows: **1** (4.52 mg), **2** (1.62 mg), **3** (3.08 mg), **4** (2.03 mg), **5** (2.42 mg), **6** (1.75 mg), **7** (2.55 mg), **8** (1.86 mg), **9** (12.59 mg), **10** (17.89 mg) and **11** (1.95 mg). The isolated pure compounds were characterized by extensive spectrometric and spectroscopic analysis by ESI‐MS or HR‐ESI‐MS, ^1^H‐NMR, ^13^C‐NMR, COSY‐NMR and HMBC‐NMR (Fig. S1–S10).

### Nematode culture

The root‐knot nematode *Meloidogyne incognita* race 3 was originally isolated from an infested field in Florida, USA. The nematode was kindly provided by Dr. D. Dickson, University of Florida, Gainesville, USA. *M. incognita* was reared on the susceptible tomato cultivar Moneymaker for 2 months in a glasshouse at 25 ± 3°C with 16‐h diurnal light and a relative humidity of 70%. Nematode eggs were extracted according to our previous work (Bogner *et al*., 2016) and collected on a 25‐μm sieve. Surface sterilization of the extracted eggs was carried out by incubating for 5 min in 0.6% (w/v) sodium hypochlorite solution under constant shaking in a rotary shaker (Edmund Bühler, Hechingen, Germany) at 100 rpm. The eggs were then washed three times with sterile water under the laminar flow hood. Further sterilization of the eggs was performed by incubating the eggs overnight, in an antibiotic solution containing 1.5 mg ml^−1^ gentamicine and 0.05 mg ml^−1^ nystatin. After the overnight incubation, sterilized eggs were rinsed three times with sterile distilled water and allowed to hatch in a modified Baermann funnel (PM 7/119 (1), 2013) at 28°C within 3–5 days to obtain second‐stage juveniles (J2s). A pinch clamp was applied on the rubber tubing allowing the collection of freshly hatched J2s that had moved though the Baermann funnel due to gravity. The nematode suspension was concentrated by transferring it to microscopy cups (‘mikroskopiernäpfe’, 40 x 40 x 16 mm: L x W x H, Labomedic, Bonn, Germany). In these microscopy cups, nematodes moved to the centre, making it easier to collect them in a small volume of water.

### Mortality bioassay

Two series of *in vitro* bioassay experiments were conducted. In the first series, the effect of the 11 isolated compounds was assayed at a concentration of 400 μg ml^−1^. For this, compounds were each dissolved in aqueous methanol (1%), and stock solutions (800 μg ml^−1^) were prepared and stored in small portions at −20°C. *In vitro* bioassay tests were performed in autoclaved microscopy cups. Microscopy cups instead of 24‐well plates were used because it was easier to observe and count the nematodes using a stereomicroscope (Leica, Wetzlar, Germany) at 8× magnification. All bioassays were carried out under aseptic conditions. The negative controls consisted of the compound solvent, 1% methanol and distilled water, while the nematicides carbofuran (Furadan) and aldicarb (Temik 10G) were used as positive controls. At the onset of the first series of experiment, the stock solution and sterile distilled water were added into each microscopy cup to achieve the required final concentration. Thereafter, an aliquot of 20 μl nematode suspension containing 50 freshly hatched J2s was added. In the end, each cup contained a total volume of 500 μl. The cups were each covered with sterile Petri dishes and carefully sealed with parafilm to avoid evaporation. The Petri dishes were incubated in the dark at 28°C. Juveniles were observed and counted under a stereomicroscope (Leica) after 24, 48 and 72 h.

Nematocidal effects were checked by transferring the dead nematodes to sterile distilled water after 72 h incubation and counting them again after 24 h. Nematodes were judged as dead if their bodies were straight with no movement. The data were transformed into the percentage mortality [mortality (%) = number of dead J2 larvae/total number of J2 larvae × 100] before statistical calculations. In this first screening, the experiment was conducted once with four replicates. This was due to the high concentrations of the compounds needed and the limited availability of the isolated pure compounds. The degree of effectiveness of each compound at 400 μg ml^−1^ after 72 h was categorized into 1–5 grades namely: 1: no effect (0% mortality), 2: poor (0–25% mortality), 3: moderate (26–50% mortality), 4: good (51–75% mortality), 5: strong (71–100% mortality).

In the second series of *in vitro* bioassay experiments, the dosage effect of the compounds on the mortality of *M. incognita* was tested using a gradient of six concentrations: 20, 50, 100, 150, 200 and 250 μg ml^−1^. The experiment was conducted twice and each treatment was replicated five times. Dose‐dependent effect allowed the calculation of the concentration of a compound which causes 50% of the nematodes to be killed (LC_50_). Compounds were considered lethal when significantly more nematodes died than in the negative control (solvent control).

### Statistical data analysis

Statistical analyses were conducted using sigma plot version 12.5. Data are shown as the mean ± S.E.M. The normal distribution (Shapiro–Wilk test) and the homogeneity of variance were checked before each analysis, and when both assumptions were met, data were further analysed via Student′s *t‐*tests (two‐group comparisons) or one‐way anova (many groups) followed by Holm–Sidak *post hoc* tests. When the data failed to meet one of the assumptions, it was further log (Log_10_x+1)‐transformed. Nonparametric tests, Mann–Whitney analysis of variance on ranks was used for data which did not satisfy one of the assumptions even after the log transformation. The number of replicates used to perform the statistical analysis for each experiment is stated in the figure or table legends, as is the specific test employed. For all data statistical significance was set at *P *≤* *0.05. LC_50_ were determined via regression analysis.

### Raman data acquisition and data processing

For Raman analysis, approximately 20 μl of the sample containing the nematodes in water was put on CaF_2_ glass slides. Raman spectra were recorded using a confocal Raman microscope (WITec Model CRM Alpha‐300R Plus; WITec GmBH, Ulm, Germany) according to Klapper *et al*. (2011) with minor modifications. Excitation (approximately 10 mW at the sample) was provided by a diode laser with a wavelength of 785 nm (Model 532; Melles Griot, Carlsbad, CA, USA). The exciting laser radiation was coupled to a Zeiss microscope (Jena, Germany) through a wavelength‐specific single‐mode optical fibre. The incident laser beam was collimated via an achromatic lens and passed through a holographic band pass filter before it was focused onto the sample through the objective of the microscope. A Nikon Fluor (60x/1.00 NA, working distance 2.0 mm) water‐immersion objective was used. The sample was scanned using a piezoelectrically driven microscope scanning stage with an x, y‐resolution of about 3 nm and a repeatability of ± 5 nm, and z‐resolution of about 0.3 nm and ± 2 nm repeatability. The sample was scanned through the laser focus in a continuous line scan at a constant stage speed of fractions of a micrometre per second. Spectra were collected at a 0.5 μm grid and an illumination time of 0.25 s, using a 300/mm grating. Raman spectra were recorded in the range of 300–3200/cm with a spectra resolution of 6/cm.

Image analysis and data processing were performed using in‐house developed spectral unmixing algorithms as detailed by Hedegaard *et al*. (2011). Further attempts were made to determine the identity of the lipids by performing mass spectrometry measurements of the crude extract‐treated samples. The obtained molecular weights from the MS peaks were searched in the Lipid MAPS^®^ Gateway website (http://www.lipidmaps.org/) and the closest hit identities were chosen.

## Author contributions

C.W.B and A.S conceived the project, took part in all experiments, conducted data analysis and wrote the manuscript; R.S.T.K analysed NMR data; G.S performed bioassay experiments; C.M and J.P performed Raman microspectroscopy analysis; D.H conducted microscopy studies and MS lipid analysis; P.P supervised the fermentation and chemical analysis; F.M.W.G wrote the grant proposal. All authors reviewed and discussed the manuscript.

## Conflict of interest

The authors declare no conflict of interests.

## Supporting information


**Table S1.** Effect of eleven Fo162 secondary metabolites on the mortality of *M. incognita*.
**Table S2.** Detected lipids in the body of *M. incognita* after treatment with the crude extract.
**Fig. S1.** General information of Gibepyrone D (*E* configuration) (1), including RT, UV and mass spectra.
**Fig. S2.** General information of Gibepyrone G (*Z* configuration) (2), including RT, UV and mass spectra.
**Fig. S3.** General information of Indole‐3‐acetic acid (3), including RT, UV and mass spectra.
**Fig. S4.** General information of 4‐Hydroxybenzoic acid (5), including RT, UV and mass spectra.
**Fig. S5.** General information of Methyl 4‐hydroxybenzoate (6) including RT, UV and mass spectra.
**Fig. S6.** General information of Methyl 2‐(4‐hydroxyphenyl)acetate (7), including RT, UV and mass spectra.
**Fig. S7.** General information of Uridine (8) including RT, UV and mass spectra.
**Fig. S8.** General information of Fusarinolic acid (9) including RT, UV and mass spectra.
**Fig. S9.** General information of Picolinic acid (10) including RT, UV and mass spectra.
**Fig. S10.** General information of Beauvericin (11) including RT, UV and mass spectra.
**Data S1.** Identification of secondary metabolites from *Fusarium oxysporum* 162.
**Data S2.** References.
