# Supplementary material for: Bioactive secondary metabolites with multiple activities from a fungal endophyte
Source: Microb Biotechnol. 2016 Dec 19;10(1):175–88. doi: 10.1111/1751-7915.12467 (PMC5270730; doi:10.1111/1751-7915.12467)
Supplement: Supplementary file 1 — Table S1. Effect of eleven Fo162 secondary metabolites on the mortality of M. incognita. Table S2. Detected lipids in the body of M. incognita after treatment with the crude extract. Fig. S1. General information of Gibepyrone D (E configuration) (1), including RT, UV and mass spectra. Fig. S2. General information of Gibepyrone G (Z configuration) (2), including RT, UV and mass spectra. Fig. S3. General information of Indole‐3‐acetic acid (3), including RT, UV and mass spectra. Fig. S4. General information of 4‐Hydroxybenzoic acid (5), including RT, UV and mass spectra. Fig. S5. General information of Methyl 4‐hydroxybenzoate (6) including RT, UV and mass spectra. Fig. S6. General information of Methyl 2‐(4‐hydroxyphenyl)acetate (7), including RT, UV and mass spectra. Fig. S7. General information of Uridine (8) including RT, UV and mass spectra. Fig. S8. General information of Fusarinolic acid (9) including RT, UV and mass spectra. Fig. S9. General information of Picolinic acid (10) including RT, UV and mass spectra. Fig. S10. General information of Beauvericin (11) including RT, UV and mass spectra. Data S1. Identification of secondary metabolites from Fusarium oxysporum 162. Data S2. References. [file MBT2-10-175-s001.docx]

**Supporting information**

**Bioactive secondary metabolites with multiple activities** **from a fungal endophyte**

**Catherine W. Bogner,^1^ Ramsay S.T. Kamdem,^2^ Gisela Sichtermann,^1^ Christian Matthäus,^3,4^** **Dirk Hölscher,^5,6^ Jürgen Popp,^3,4^ Peter Proksch,^2^ Florian M.W. Grundler,^1^ Alexander Schouten^1,7^ ⃰**

^1^Institute of Crop Science and Resource Conservation (INRES), Department of Molecular Phytomedicine, University of Bonn, Karlrobert-Kreiten Str. 13, 53115 Bonn, Germany.

^2^Institute of Pharmaceutical Biology and Biotechnology, Heinrich-Heine-University Düsseldorf, Universitäts Str. 1, Building. 26.23, 40225 Düsseldorf, Germany.

^3^Institute of Photonic Technology, Workgroup Spectroscopy/Imaging, Albert-Einstein-Str. 9, 07745 Jena, Germany.

^4^Institute of Physical Chemistry and Abbe Center of Photonics, Friedrich Schiller University, Helmholtzweg 4, 07743 Jena, Germany

^5^Research Group Biosynthesis/NMR, Max Planck Institute for Chemical Ecology, Hans-Knöll-Str. 8, 07745 Jena, Germany.

^6^Present address: Organic Plant Production and Agroecosystems Research in the Tropics and Subtropics (OPATS), University of Kassel, Steinstr. 19, 37213 Witzenhausen, Germany.

^7^Present address: Laboratory of Nematology, Wageningen University, Droevendaalsesteeg 1, 6708 PD Wageningen, The Netherlands.

**Corresponding author:**

Alexander Schouten

Laboratory of Nematology, Wageningen University

Droevendaalsesteeg 1, 6708 PD Wageningen

Tel: +31-627-500757

[sander.schouten@hotmail.com](mailto:sander.schouten@hotmail.com)

Table of Contents

Table [S1. Effect of eleven Fo162 secondary metabolites on the mortality of *M. incognita* 2](#_Toc459470620)

Table [S2. Detected lipids in the body of *M. incognita* after treatment with the crude extract 3](#_Toc459470621)

Data [S1. Identification of secondary metabolites from *Fusarium oxysporum* 162 4](#_Toc459470622)

Figure [S1. General information of Gibepyrone D (*E* configuration) (1), including RT, UV and mass spectra 7](#_Toc459470623)

Figure [S1.1. ^1^H NMR (600 MHz, Methanol-*d4*) spectrum of compound 1 (Gibepyrone D) 8](#_Toc459470624)

[S1.2. COSY NMR (600 MHz, Methanol-*d4*) spectrum of compound 1 (Gibepyrone D) 8](#_Toc459470625)

[S1.3. HMBC NMR (600 MHz, Methanol-*d4*) spectrum of compound 1 (Gibepyrone D) 9](#_Toc459470626)

Figur [S2. General information of Gibepyrone G (*Z* configuration) (2), including RT, UV and mass spectra 10](#_Toc459470627)

[S2.1. ^1^H NMR (600 MHz, Methanol-*d4*) spectrum of compound 2 (Gibepyrone G) 11](#_Toc459470628)

Figure [S3. General information of Indole-3-acetic acid (3), including RT, UV and mass spectra 12](#_Toc459470629)

[S3.1. ^1^H NMR (600 MHz, Methanol-d4) spectrum of compound 3 (Indole-3-acetic acid) 13](#_Toc459470630)

[S3.2. COSY NMR (600 MHz, Methanol-*d4*) spectrum of compound 3 (Indole-3-acetic acid) 13](#_Toc459470631)

[S3.3. ROESY NMR (600 MHz, Methanol-*d4*) spectrum of compound 3 (Indole-3-acetic acid) 14](#_Toc459470632)

Figure [S4. General information of 4-Hydroxybenzoic acid (5), including RT, UV and mass spectra 15](#_Toc459470633)

[S4.1. ^1^H NMR (600 MHz, Methanol-d4) spectrum of compound 5 (4-Hydroxybenzoic acid) 16](#_Toc459470634)

[S4.2. COSY NMR (600 MHz, Methanol-*d4*) spectrum of compound 5 (4-Hydroxybenzoic acid) 16](#_Toc459470635)

Figure [S5. General information of Methyl 4-hydroxybenzoate (6) including RT, UV and mass spectra 17](#_Toc459470636)

[S5.1. ^1^H NMR (600 MHz, Methanol-d4) spectrum of compound 6 (Methyl 4-hydroxybenzoate) 18](#_Toc459470638)

[S5.2. COSY NMR (600 MHz, Methanol-*d4*) spectrum of compound 6 (Methyl 4-hydroxybenzoate) 18](#_Toc459470639)

Figure [S6. General information of Methyl 2-(4-hydroxyphenyl)acetate (7), including RT, UV and mass spectra 19](#_Toc459470640)

[S6.1. ^1^H NMR (600 MHz, Methanol-d4) spectrum of compound 7 (Methyl 2-(4-hydroxyphenyl)acetate) 20](#_Toc459470641)

[S6.2. COSY NMR (600 MHz, Methanol-*d4*) spectrum of compound 7 (Methyl 2-(4-hydroxyphenyl)acetate) 20](#_Toc459470642)

Figure [S7. General information of Uridine (8) including RT, UV and mass spectra 21](#_Toc459470643)

[S7.1. ^1^H NMR (600 MHz, Methanol-d4) spectrum of compound 8 (Uridine) 22](#_Toc459470644)

[S7.2. COSY NMR (600 MHz, Methanol-*d4*) spectrum of compound 8 (Uridine) 22](#_Toc459470645)

Figure [S8. General information of Fusarinolic acid (9) including RT, UV and mass spectra 23](#_Toc459470646)

[S8.1. ^1^H NMR (300 MHz, Methanol-d4) spectrum of compound 9 (Fusarinolic acid) 24](#_Toc459470647)

[S8.2. COSY NMR (600 MHz, Methanol-*d4*) spectrum of compound 9 (Fusarinolic acid) 24](#_Toc459470648)

[S8.3. HMBC NMR (600 MHz, Methanol-*d4*) spectrum of compound 9 (Fusarinolic acid) 25](#_Toc459470649)

Figure [S9.General information of Picolinic acid (10) including RT, UV and mass spectra 26](#_Toc459470650)

[S9.1. ^1^H NMR (300 MHz, Methanol-d4) spectrum of compound 10 (Picolinic acid) 27](#_Toc459470651)

[S9.2. COSY NMR (300 MHz, Methanol-*d4*) spectrum of compound 10 (Picolinic acid) 27](#_Toc459470652)

[S9.3. HMBC NMR (300 MHz, Methanol-*d4*) spectrum of compound 10 (Picolinic acid) 28](#_Toc459470653)

Figure [S10. General information of Beauvericin (11) including RT, UV and mass spectra 29](#_Toc459470654)

[S10.1. ^1^H NMR (300 MHz, Methanol-d4) spectrum of compound 11 (Beauvericin) 30](#_Toc459470655)

[S10.2. COSY NMR (300 MHz, Methanol-*d4*) spectrum of compound 11 (Beauvericin) 30](#_Toc459470656)

[S10.3. HMBC NMR (300 MHz, Methanol-*d4*) spectrum of compound 11 (Beauvericin) 31](#_Toc459470657)

Data [S2. References 32](#_Toc459470658)

# Table S1. Effect of eleven Fo162 secondary metabolites on the mortality of *M. incognita*

| **Table S1:** Effect of eleven Fo162 secondary metabolites at 400 µg/ml on the mortality of *Meloidogyne incognita* J2 larvae after 24, 48 and 72 hours of contact. | | | | | |
| --- | --- | --- | --- | --- | --- |
|  |  | **Mortality (%)** | | |  |
| **ID Code*** | **Compound** | **24 h** | **48 h** | **72 h** | **Lethality at 72 h**** |
| **1** | Gibepyrone D: *E* configuration | 56.0 ±1.8^c^ | 75.0 ± 2.1^c^ | 85.5 ±2.6^b^ | Strong |
| **2** | Gibepyrone G: *Z* configuration | 19.5 ±3.6^ef^ | 27.0 ± 3.1^f^ | 37.5 ±2.9^e^ | Moderate |
| **3** | Indole-3-acetic acid | 66.5 ±1.7^b^ | 77 ± 2.5^c^ | 86.0 ± 1.8^b^ | Strong |
| **4** | Indole-3-acetic acid methyl ester | 23.0 ±2.4^e^ | 32.5 ± 2.2^e^ | 45.5 ± 2.2^d^ | Moderate |
| **5** | 4-Hydroxybenzoic acid | 71.5 ±1.7^a^ | 84 ± 2.1^b^ | 97.0 ± 1.3^a^ | Strong |
| **6** | Methyl 4-hydroxybenzoate | 22.5 ±2.2^e^ | 35 ± 1.7^e^ | 38.0 ±2.6^e^ | Moderate |
| **7** | Methyl 2-(4-hydroxyphenyl)acetate | 31.5 ±1.7^d^ | 43.5 ± 1.7^d^ | 58.0 ± 2.1^c^ | Good |
| **8** | Uridine | ― | ― | ― | None |
| **9** | Fusarinolic acid | 7 ±2.4^g^ | 16.5 ± 1.7^g^ | 21.5 ±1.3^f^ | Poor |
| **10** | 5-(But-3-en-1-yl)picolinic acid | 13.5 ±1.7^f^ | 17.0 ± 1.9^g^ | 22.0 ±1.4^f^ | Poor |
| **11** | Beauvericin | ― | ― | ― | None |
| **NC** | 1% Methanol | 4±0.7^g^ | 9.7±2.1^h^ | 12.1±1.9^g^ | Poor |
| **P1** | Carbofuran | 70.5 ±1.7^a^ | 92.0 ± 1.6^a^ | 99.5 ±0.5^a^ | Strong |
| **P2** | Aldicarb | 35.7 ±2.6^d^ | 40.5 ± 2.2^d^ | 44.1 ±2.2^d^ | Moderate |
|  | | | | | |

**1-11**: compound code number as described in Fig. S1; **NC**: negative control; **P1** and **P2**: positive controls**.****The degree of effectiveness of each compound at 400 µg/ml after 72 h was divided into 5 categories, namely: No effect (0% death); Poor (0-25% death); Moderate (26-50% death); Good (51-75% death); and Strong (71-100% death).
A compound was considered lethal when it caused a significantly (*P≤*0.05) higher percentage of nematode to die after 24, 48 and 72 hours than the **NC**. Data are expressed as the means ± standard errors of four replicates. Significance was tested according to Holm-Sidak multiple comparisons versus control group using Sigma plot 12.5. Means followed by the same letter are not significantly different from the mean percentage of dead nematodes in the negative control.

# Table S2. Detected lipids in the body of *M. incognita* after treatment with the crude extract

| **Table S2:** Detected lipids in the body of *M. incognita* after treatment with the crude extract | | | | | | | |
| --- | --- | --- | --- | --- | --- | --- | --- |
|  |  |  |  |  |  |  |  |
| **No.** | **Category** | **Main Class** | **MW** | **H+**  **Adduct** | **Found**  **In LM** | **Hits** | **LM_ID** |
| 1 | Unknown | Unknown | 335.1758 | 336.1758 | ― | ― | ― |
| 2 | Prenol lipids | Isoprenoids | 410.2207 | 411.2207 | 410.2305 | 75 | LMPR0104030004 |
| 3 | Glycerophospholipids | Glycerophosphocholines | 465.3259 | 466.3259 | 465.3219 | 10 | LMGP01070003 |
| 4 | Polyketides | Flavonoids | 516.2010 | 516.2010 | 516.1784 | 25 | LMPK12120065 |
| 5 | Glycerophospholipids | Glycerophosphates | 648.4474 | 648.4474 | 648.4730 | 33 | LMGP10010012 |
| 6 | Glycerophospholipids | Glycerophosphocholines | 675.4696 | 675.4696 | 675.4839 | 30 | LMGP01010392 |
| 7 | Glycerophospholipids | Glycerophosphoserines | 703.4981 | 703.4981 | 703.4424 | 43 | LMGP03010053 |
| 8 | Polyketides | Polyenes | 913.6940 | 914.6940 | 913.5551 | 3 | LMGP03010053 |
| 9 | Glycerophospholipids | Glycerophosphoserines | 903.7094 | 904.7094 | 903.6928 | 5 | LMGP03010943 |
| 10 | Unknown | Unknown | 967.7431 | 968.7431 | ― | ― | ― |
| 11 | Unknown | Unknown | 995.7742 | 996.7731 | ― | ― | ― |
| 12 | Unknown | Unknown | 965.7742 | 966.7742 | ― | ― | ― |
| 13 | Prenol lipids | Isoprenoids | 594.3653 | 595.3653 | 594.3703 | 135 | LMPR01070051 |
| 14 | Glycero lipids | Diradylglyceros | 602.5338 | 603.5338 | 602.5638 | 22 | LMGL02030027 |
| 15 | Unknown | Unknown | 613.4349 | 614.4349 | ― | ― | ― |
| 16 | Sphingolipids | Ceramides | 617.4283 | 618.4283 | 617.4283 | 2 | LMSP02050002 |
| 17 | Glycero lipids | Diradylglyceros | 629.4296 | 630.4296 | 629.6002 | 1 | LMGL02010318 |
| 18 | Glycerophospholipids | Glycerophosphates | 634.4314 | 635.4314 | 634.4574 | 27 | LMGP10010055 |
| 19 | Sphingolipids | Neutral glycosphingolipids | 641.4280 | 642.4280 | 641.4867 | 9 | LMSP0501AA52 |
| 20 | Glycerophospholipids | Glycerophosphates | 648.4474 | 649.4474 | 648.4730 | 33 | LMGP10010012 |
| 21 | Glycerophospholipids | Glycerophosphoethanolamines | 657.4614 | 658.4614 | 657.4614 | 5 | LMGP02010367 |
| 22 | Glycerophospholipids | Glycerophosphates | 670.4688 | 671.4688 | 670.4574 | 32 | LMGP10010102 |
| 23 | Glycerophospholipids | Glycerophosphocholines | 675.4696 | 676.4696 | 675.4839 | 30 | LMGP01010392 |
| 24 | Glycerophospholipids | Glycerophosphothanolamines | 685.4544 | 686.4544 | 685.4683 | 14 | LMGP02010374 |
| 25 | Glycerophospholipids | Glycerophosphoserines | 691.4667 | 692.4667 | 691.4424 | 41 | LMGP03010051 |
| 26 | Glycerophospholipids | Glycerophosphoethanolamines | 695.4393 | 695.4393 | 695.4526 | 12 | LMGP02010400 |
| 27 | Glycerophospholipids | Glycerophosphoserines | 703.4981 | 704.4981 | 703.4788 | 43 | LMGP03030006 |
| 28 | Glycerophospholipids | Glycerophosphocholines | 713.4855 | 714.4855 | 713.4855 | 27 | LMGP01011349 |
| 29 | Glycerophospholipids | Glycerophosphoserines | 731.5282 | 732.5282 | 731.5101 | 65 | LMGP03020009 |
| 30 | Glycerophospholipids | Glycerophosphates | 746.3891 | 747.3891 | 746.4887 | 55 | LMGP10010040 |
| 31 | Glycerophospholipids | Glycerophosphoethanolamines | 759.3969 | 760.3969 | 759.4839 | 77 | LMGP02010703 |
| 32 | Glycerophospholipids | Glycerophosphoserines | 771.5230 | 772.5230 | 771.5050 | 61 | LMGP0310153 |
| 33 | Glycerophospholipids | GlycerophosphoGlyceros | 784.4979 | 785.4979 | 784.5254 | 58 | LMGP04010006 |
| 34 | Glycerophospholipids | Glycerophosphoinositols | 794.4828 | 795.4828 | 794.4945 | 51 | LMGP0610006 |
| 35 | Glycerophospholipids | Glycerophosphoserines | 801.4169 | 802.4169 | 801.4581 | 73 | LMGP03010449 |
| 36 | Glycerophospholipids | Glycerophosphoinositols | 810.3438 | 811.3438 | 810.5238 | 56 | LMGP06010007 |
| 37 | Glycerophospholipids | Glycerophosphoinositols | 820.4620 | 821.4620 | 821.5102 | 69 | LMGP06010052 |
| 38 | Glycerophospholipids | Glycerophosphoinositols | 826.5073 | 827.5073 | 826.4632 | 71 | LMGP06010100 |
| 39 | Glycerophospholipids | Glycerophosphoinositols | 834.3450 | 835.3450 | 834.5258 | 69 | LMGP06010034 |
| 40 | Glycerophospholipids | Glycerophosphoinositols | 846.4745 | 847.4745 | 846.5258 | 90 | LMGP06010121 |
| 41 | Glycerophospholipids | Glycerophosphocholines | 857.5966 | 858.5966 | 857.5935 | 31 | LMGP01011865 |
| 42 | Sphingolipids | Phosphosphingolipids | 869.6001 | 870.6001 | 869.5993 | 24 | LMSP03030038 |
| 43 | Glycerophospholipids | Glycerophosphocholines | 877.6750 | 878.6750 | 877.6924 | 6 | LMGP01020278 |
| 44 | Glycerophospholipids | Glycerophosphoserines | 885.6673 | 886.6673 | 885.6459 | 10 | LMGP03010700 |
| 45 | Glycerophospholipids | Glycerophosphoserines | 899.6821 | 900.6821 | 899.6651 | 9 | LMGP03010723 |
| 46 | Glycerophospholipids | Glycerophosphoserines | 899.6821 | 900.6821 | 899.6651 | 9 | LMGP03010723 |
| 47 | Sphingolipids | Phosphosphingolipids | 909.6635 | 910.6635 | 909.6670 | 9 | LMSP03030016 |
| 48 | Polyketides | Polyenes | 913.6940 | 914.6940 | 913.5551 | 3 | LMPK06000003 |
| 49 | Glycerophospholipids | Glycerophosphocholines | 927.7106 | 928.7106 | 927.7656 | 1 | LMGP01011105 |
| 50 | Unknown | Unknown | 935.6791 | 936.6791 | ― | ― | ― |
| 51 | Sphingolipids | Phosphosphingolipids | 937.6929 | 938.6929 | 937.6983 | 8 | LMSP03030017 |
| 52 | Unknown | Unknown | 939.7094 | 940.7094 | ― | ― | ― |
| 53 | Unknown | Unknown | 941.7213 | 942.7213 | ― | ― | ― |
| 54 | Unknown | Unknown | 951.7053 | 952.7053 | ― | ― | ― |
| 55 | Unknown | Unknown | 959.6709 | 960.6709 | ― | ― | ― |
| 56 | Unknown | Unknown | 961.6898 | 962.6898 | ― | ― | ― |
| 57 | Glycerophospholipids | Glycerophosphoethanolamines | 963.7118 | 964.7118 | 963.7656 | 13 | LMGP02010035 |
| 58 | Unknown | Unknown | 967.7431 | 968.7431 | ― | ― | ― |
| 59 | Sphingolipids | Phosphosphingolipids | 987.7077 | 988.7077 | 987.6259 | 4 | LMSP03030055 |
| 60 | Unknown | Unknown | 995.7742 | 966.7742 | ― | ― | ― |
| No. 1-12: Lipids measured in the molecular weight range of 0-1300, No. 13-28: 600-700, No: 28-45: 700-900 and No: 46-60: 900-1000. Using the molecular weights, the possible identity of the lipids was searched in Lipid maps website: <http://www.lipidmaps.org/> | | | | | | | |

# Data S1. Identification of secondary metabolites from *Fusarium oxysporum* 162

Compound **1** was isolated as a white powder and its molecular formula and mass were assigned as C_10_H_10_O_4_, [M+H]^+^ 195.1 from ESI-MS and NMR spectra. The results of ^1^H-NMR spectrum show two signals at 6.70 and 7.40 ppm due to two vicinal protons in a 3,6-disubsituted α-pyrone system. The most deshielded hydrogen, H-2, is in β position to the carbonyl group and shows a long range coupling with the methyl at 2.36 ppm. The isopropenyl residue, located on C-6, bears the methyl group at 2.11 ppm and the olefinic proton at 6.62 ppm. The data was confirmed by a 2D NMR ^1^H/^1^H homonuclear correlation while the careful analysis of ^13^C NMR spectra also confirmed the presence of 3-methyl-6-(7-methylpropenyl)-α–pyrone. The configuration of the double bond was assigned to be cis due to the absence of long range NOESY correlation between proton H-8 and CH_3_-7. Compound **2** depicts a similar mass, UV and NMR pattern as compound **1**. Differences were observed in the NOESY correlation between proton H-8 and CH_3_-7. This correlation indicates that compounds **1** and **2** are in *E* and *Z* configuration respectively and can be identified as gibepyrone D and G. The data is in accordance with those previously reported (Barrero *et al*., 1993).

Compound **3** was obtained as a yellow powder. ESI-MS and NMR measurements gave the molecular formula C_10_H_9_NO_2_, [M+H]^+^, 176.0. The UV absorption at 218.4 nm and 279.6 nm showed typical values of indole-3-substituted derivatives. Analysis of ^1^H- and ^13^C-NMR data indicated the presence of an acetyl hydroxide moiety at 3.73/172.8 ppm in addition to the indole unit. The positions of acetyl hydroxide group were deduced by HMBC correlations of H-9 with C-1/C-8 positions. Compound **4** has a similar spectrum with compound **3** except that compound **4** possess an ester methoxy group which appeared as a singlet at 3.68 ppm in the ^1^H NMR analysis and has an HMBC correlation with the carbonyl group at 172.8 ppm. Compound **4** is therefore an acetate of **3**. The spectral data enabled the identification of the compounds as indol-3-acetic acid **3** and indol-3-acetic acid methyl ester **4** respectively. The data is in agreement with literature (Evidente *et al*., 1993).

Compound **5** was isolated as a yellow powder. Its molecular formula was determined to be C_7_H_6_O_3_ and the mass was found to be 139.4 [M+H]^+^ inferred from the ESI-MS and NMR measurements corresponding to five degrees of unsaturation. The ^1^H-NMR spectrum shows a typical AA’BB’ spin system at 7.06, 7.04, 6.71 and 6.70 ppm corresponding to a *para* - disubstituted benzene ring, this was confirmed by the UV (λ_max_) absorption at 229.4 and 276.0 nm and available literature comparison (Cho *et al*., 1998). The HMBC correlation shows that H-3 and H-5 are correlated with a carbonyl group at 169.3 ppm. The data confirmed that compound **5** is 4-hydroxybenzoic acid. The ESI-MS of compound **6** exhibited the molecular formula C_8_H_8_O_3_ which was derived from the molecular ion [M+H]^+^ at m/z 152.8 and NMR data, corresponding to five degrees of unsaturation. The NMR data of **5** were closely related to those of **6**, except for the presence of the ester methoxy at 3.36 ppm in **6** which shows the HMBC correlation with a carbonyl group at 165.5 ppm. This indicates that compound **6** is acetate of compound **5** and therefore can be assigned to methyl 4-hydroxybenzoate.

Compound **7** was isolated as yellow oil and the molecular formula was assigned as C_9_H_10_O_3_ (five degrees of unsaturation) according to mass spectrometry and NMR analyses. The molecular mass 166.8 [M+H]^+^ was determined from the ESI-MS calculations. The ^1^H-NMR proton spectra shows a typical AA’BB’ spin system at 7.08, 7.06, 6.72, and 6.71 ppm corresponding to a *para*- disubstituted benzene ring. Additionally, a benzylic methylene at 3.52 ppm and a methyl ester at 3.66 ppm were evident. The HMBC spectrum showed a correlation of OCH_3_ with a carbonyl at 174.8 ppm (C-8) and H-7 with 130.3 ppm (C-3)/ 130.3 ppm (C-5). On the basis of all the foregoing evidence, **7** was found to be methyl 2-(4-hydroxyphenyl)acetate. The data is in accordance with those previously reported (Li *et al*., 2015).

Compound **8** was isolated as a white powder. It´s molecular formula C_9_H_12_N_2_O_6_ (five degrees of unsaturation) was derived from the molecular ion [M+H]^+^ at m/z 244.8 in the ESI-MS and NMR meaurements. The UV (λ_max_) absorption at 218.2 and 280.3 nm indicated the presence of an uracil ring. The^1^H-NMR spectra shows an AB spin system at 8.01 ppm and 5.70 ppm. The isolated spin system contains hydroxylmethines at 5.90 ppm, 4.00 ppm, 3.84 ppm, 3.73 ppm and prochiral hydroxymethylene at 4.18 ppm, 4.15 ppm attributed to the furanoside moiety. The HMBC spectrum shows a correlation of H-2 with C-1’/C-4/C-5. The above data and those of ^13^C NMR were in agreement with those of uridine (Li *et al*., 2005).

Compound **9** was obtained as a white amorphous powder. Its molecular formula was established as C_10_H_13_NO_3_, 195.8 [M+H]^+^ by ESI-MS and NMR measurements, indicating ﬁve degrees of unsaturation. The UV spectrum showed characteristic peaks of fusaric acid analogues at UV (λ_max_) 229.4 and 276.0 nm (Liu *et al*., 2016). The ^1^H NMR spectrum exhibited a typical ABX coupling system at 8.25, 8.19 and 7.96 ppm suggesting the presence of a 2,5-substituted pyridine ring, which was conﬁrmed by the HMBC correlations from H-3 to C-5 (140.8 ppm), from H-4 to C-2 (147.2 ppm) and C-6 (148.5 ppm), from H-6 to C-2 and C-4 (132.2 ppm). The proton spectra and COSY also showed an additional spin system at H_2_-7 (2.75 ppm)/H_2_-8 (1.71 ppm), H2-8/H_1_-9 (3.65 ppm) and H1-9/ H_3_-10 (1.18 ppm) attributed to a butyl group. The presence of one signal at H-9, 3.65 ppm /74.8 ppm suggested the presence of an oxymethine on the butyl group. The positions of the acid, butyl group and hydroxyl group were deduced by the HMBC correlations H-3 with C-11/C-8, H-7 with C-4/C-6/C-9. Finally, it was identified as fusarinolic acid. The HRESIMS spectrum of 5-(but-3-en-1-yl)picolinic acid, **10** indicated the molecular formula C_10_H_11_NO_2_, and a molecular weight of 178.0862 which seems to be a product of deshydratation of **9**. The NMR data were closely related to those of **9**, except the presence of signals of a terminal double bond at 5.76 ppm, H-9/114.7 ppm and 4.94 ppm, H-10/102.5 ppm in **10**. The presence in COSY spectrum of the spin system H-10 – H-9 – H-8 indicates that 5-(but-3-en-1-yl)picolinic acid 10 is a dehydratation product of **9.**

Compound **11** was isolated as a white powder. Its molecular formula of C_45_H_58_N_3_O_9_ was derived from the molecular ion [M+H]^+^ at m/z 784.4201 (calcd 784,4173) in the HR-ESI-MS, corresponding to nineteen degrees of unsaturation. The UV (λ_max_) at 216 and 258 nm is consistent with a monosubstituted benzene ring. The simplicity of the ^1^H NMR is indicative of its symmetric and cyclic nature.

The ^1^H NMR spectrum exhibited three methyls at 3.17 ppm, each linked to nitrogen, two methyls at 0.87 and 0.27 ppm that are coupling together with a methine at 1.83 ppm thereby corresponding to an isopropyl group. Three α-amino protons at 5.82 ppm suggest that the compound has a peptide structure. Detailed interpretation of the COSY and HMBC spectra revealed the presence of 2 amino acid residues, namely N-methyl-[phenylalanyl](https://en.wikipedia.org/wiki/L-phenylalanine) and hydroxy-iso-valeryl. The data is in accordance with those of beauvericin found in the literature (Hamill *et al*., 1969)

# Figure S1. General information of Gibepyrone D (*E* configuration) (1), including RT, UV and mass spectra

| **Compound 1: Gibepyrone D** | |
| --- | --- |
| Synonyms | *(E)* -3-(3-methyl-2-oxo-2H-pyran-6-yl)but-2-enoic acid |
| Sample code | 7FOSeph4 |
| Biological source | *Fusarium oxysporum* 162 |
| Sample amount | 4.52 mg |
| Physical description | Colorless cyrstalline |
| Molecular formula | C_10_H_10_O_4_ |
| Molecular weight | 194 g/mol |
| Retention time (HPLC) | 27.85 min |
|  | |
|  | |

## Figure S1.1. ^1^H NMR (600 MHz, Methanol-*d4*) spectrum of compound 1 (Gibepyrone D)

## Figure S1.2. COSY NMR (600 MHz, Methanol-*d4*) spectrum of compound 1 (Gibepyrone D)

## Figure S1.3. HMBC NMR (600 MHz, Methanol-*d4*) spectrum of compound 1 (Gibepyrone D)

# Figure S2. General information of Gibepyrone G (*Z* configuration) (2), including RT, UV and mass spectra

| **Compound 2: Gibepyrone G** | |
| --- | --- |
| Synonyms | *(Z)* -3-(3-methyl-2-oxo-2H-pyran-6-yl)but-2-enoic acid |
| Sample code | 6FOSeph4 |
| Biological source | *Fusarium oxysporum* 162 |
| Sample amount | 1.62 mg |
| Physical description | Colorless cyrstalline |
| Molecular formula | C_10_H_10_O_4_ |
| Molecular weight | 194 g/mol |
| Retention time (HPLC) | 30.77 min |
|  | |
|  | |

## Figure S2.1. ^1^H NMR (600 MHz, Methanol-*d4*) spectrum of compound 2 (Gibepyrone G)

# Figure S3. General information of Indole-3-acetic acid (3), including RT, UV and mass spectra

| **Compound 3: Indol-3-acetic acid** | |
| --- | --- |
| Synonyms | 2-(1*H*-Indol-3-yl)acetic acid |
| Sample code | 1.6FOSeph6aSP |
| Biological source | *Fusarium oxysporum* 162 |
| Sample amount | 3.08 mg |
| Physical description | Yellow solid |
| Molecular formula | C_10_H_9_NO_2_ |
| Molecular weight | 175 g/mol |
| Retention time (HPLC) | 26.68 min |
|  | |
|  | |

## Figure S3.1. ^1^H NMR (600 MHz, Methanol-d4) spectrum of compound 3 (Indole-3-acetic acid)

## Figure S3.2. COSY NMR (600 MHz, Methanol-*d4*) spectrum of compound 3 (Indole-3-acetic acid)

## Figure S3.3. ROESY NMR (600 MHz, Methanol-*d4*) spectrum of compound 3 (Indole-3-acetic acid)

# Figure S4. General information of 4-Hydroxybenzoic acid (5), including RT, UV and mass spectra

| **Compound 5: 4-Hydroxybenzoic acid** | |
| --- | --- |
| Synonyms | 4-Hydroxybenzoic acid |
| Sample code | 1aPeak1upaTLC2SPof 2.5FOSeph5 |
| Biological source | *Fusarium oxysporum* 162 |
| Sample amount | 2.42 mg |
| Physical description | Yellow solid |
| Molecular formula | C_7_H_6_O_3_ |
| Molecular weight | 138 g/mol |
| Retention time (HPLC) | 21.10 min |
|  | |
|  | |

## Figure S4.1. ^1^H NMR (600 MHz, Methanol-d4) spectrum of compound 5 (4-Hydroxybenzoic acid)

## Figure S4.2. COSY NMR (600 MHz, Methanol-*d4*) spectrum of compound 5 (4-Hydroxybenzoic acid)

# Figure S5. General information of Methyl 4-hydroxybenzoate (6) including RT, UV and mass spectra

| **Compound 6: Methyl 4-hydroxybenzoate** | |
| --- | --- |
| Synonyms | Methyl 4-hydroxybenzoate |
| Sample code | 2aPeak1DownaTLC2SPof 2.5FOSeph5 |
| Biological source | *Fusarium oxysporum* 162 |
| Sample amount | 1.75 mg |
| Physical description | Yellow crystalline |
| Molecular formula | C_8_H_8_O_3_ |
| Molecular weight | 152 g/mol |
| Retention time (HPLC) | 16.57 min |
|  | |
|  | |

## Figure S5.1. ^1^H NMR (600 MHz, Methanol-d4) spectrum of compound 6 (Methyl 4-hydroxybenzoate)

## Figure S5.2. COSY NMR (600 MHz, Methanol-*d4*) spectrum of compound 6 (Methyl 4-hydroxybenzoate)

# Figure S6. General information of Methyl 2-(4-hydroxyphenyl)acetate (7), including RT, UV and mass spectra

| **Compound 7: Methyl 2-(4-hydroxyphenyl)acetate** | |
| --- | --- |
| Synonyms | Methyl 2-(4-hydroxyphenyl)acetate |
| Sample code | 4SPof 2.5FOSeph5aAC |
| Biological source | *Fusarium oxysporum* 162 |
| Sample amount | 2.55 mg |
| Physical description | Yellow oil |
| Molecular formula | C_9_H_10_O_3_ |
| Molecular weight | 166 g/mol |
| Retention time (HPLC) | 34.14 min |
|  | |
|  | |

## Figure S6.1. ^1^H NMR (600 MHz, Methanol-d4) spectrum of compound 7 (Methyl 2-(4-hydroxyphenyl)acetate)

## Figure S6.2. COSY NMR (600 MHz, Methanol-*d4*) spectrum of compound 7 (Methyl 2-(4-hydroxyphenyl)acetate)

# Figure S7. General information of Uridine (8) including RT, UV and mass spectra

| **Compound 8: Uridine** | |
| --- | --- |
| Synonyms | 1-(3,4-dihydroxymethyl)tetrahydrofuran-2-yl)pyrimidine-2,4 (1 *H*, *3H*)-dione |
| Sample code | 5.5FOSeph5aACaSP |
| Biological source | *Fusarium oxysporum* 162 |
| Sample amount | 1.86 mg |
| Physical description | Colourless crystalline |
| Molecular formula | C_9_H_12_N_2_O_6_ |
| Molecular weight | 244 g/mol |
| Retention time (HPLC) | 9.07 min |
|  | |
|  | |

## Figure S7.1. ^1^H NMR (600 MHz, Methanol-d4) spectrum of compound 8 (Uridine)

## Figure S7.2. COSY NMR (600 MHz, Methanol-*d4*) spectrum of compound 8 (Uridine)

# Figure S8. General information of Fusarinolic acid (9) including RT, UV and mass spectra

| **Compound 9:** **Fusarinolic acid** | |
| --- | --- |
| Synonyms | 5-(3-hydroxybutyl)picolinic acid |
| Sample code | 2aPeak2MiddleTLCofFO-RP-18-3 |
| Biological source | *Fusarium oxysporum* 162 |
| Sample amount | 12.59 mg |
| Physical description | Yellow solid |
| Molecular formula | C_10_H_13_NO_3_ |
| Molecular weight | 195 g/mol |
| Retention time (HPLC) | 9.89 min |
|  | |
|  | |

## Figure S8.1. ^1^H NMR (300 MHz, Methanol-d4) spectrum of compound 9 (Fusarinolic acid)

#

## Figure S8.2. COSY NMR (600 MHz, Methanol-*d4*) spectrum of compound 9 (Fusarinolic acid)

## Figure S8.3. HMBC NMR (600 MHz, Methanol-*d4*) spectrum of compound 9 (Fusarinolic acid)

#

# Figure S9. General information of Picolinic acid (10) including RT, UV and mass spectra

| **Compound 10:** **Picolinic acid** | |
| --- | --- |
| Synonyms | 5-(but-3-en-1-yl)picolinic acid |
| Sample code | 2d Peak2 down FO-RP-18-4aTLC |
| Biological source | *Fusarium oxysporum* 162 |
| Sample amount | 17.89 mg |
| Physical description | Yellow solid |
| Molecular formula | C_10_H_11_NO_2_ |
| Molecular weight | 177 g/mol |
| Retention time (HPLC) | 16.70 min |
|  | |
|  | |

## Figure S9.1. ^1^H NMR (300 MHz, Methanol-d4) spectrum of compound 10 (Picolinic acid)

## Figure S9.2. COSY NMR (300 MHz, Methanol-*d4*) spectrum of compound 10 (Picolinic acid)

## Figure S9.3. HMBC NMR (300 MHz, Methanol-*d4*) spectrum of compound 10 (Picolinic acid)

##

# Figure S10. General information of Beauvericin (11) including RT, UV and mass spectra

| **Compound 11: Beauvericin** | |
| --- | --- |
| Synonyms | (3S,6R,9S,12R,15S,18R)-3,9,15-tribenzyl-6,12,18-triisopropyl-4,10,16-trimethyl-1,7,13-trioxa-4,10,16-triazacyclooctadecane-2,5,8,11,14,17-hexaone |
| Sample code | FoSeph2-N2-1 |
| Biological source | *Fusarium oxysporum* 162 |
| Sample amount | 1.95 mg |
| Physical description | White crystals |
| Molecular formula | C_45_H_57_N_3_O_9_ |
| Molecular weight | 783 g/mol |
| Retention time (HPLC) | 33.39 min |
|  | |
|  | |

## Figure S10.1. ^1^H NMR (300 MHz, Methanol-d4) spectrum of compound 11 (Beauvericin)

## Figure S10.2. COSY NMR (300 MHz, Methanol-*d4*) spectrum of compound 11 (Beauvericin)

## Figure S10.3. HMBC NMR (300 MHz, Methanol-*d4*) spectrum of compound 11 (Beauvericin)

# Data S2. References

Barrero, A.F., Oltra, J.E., Herrador, M.M., Cabrera, E., Juan, F.S., Quilez, J.F., *et al*. (1993) Gibepyrones: α-pyrones from *Gibberella fujikuroi*. *Tetrahedron* **49:**141–150.

Cho, J.-Y., Moon, J.-H., Seong, K.-Y. and Park, K.-H. (1998) Antimicrobial activity of 4-hydroxybenzoic acid and trans 4-hydroxycinnamic acid isolated and identified from rice hull. *Biosci, Biotechnol Biochem* **62:** 2273–2276.

Evidente, A., Iacobellis, N.S and Sisto, A. (1993) Isolation of indole-3-acetic acid methylester, a metabolite of indole-3-acetic acid from *Pseudomonas amygdali*. *Experientia* **49:** 182–183.

Hamill, R.L., Higgens, C.E., Boaz, M.E & Gorman, M. (1969) The structure of beauvericin, a new despsipeptide antibiotic toxic to *Artemia salina*. *Tetrahedron Lett* **49:** 4255–4258.

Li, W., Yang, X., Yang, Y., and Ding, Z. (2015) A new natural nucleotide and other antibacterial metabolites from an endophytic Nocardia sp. *Nat Prod Res.* **29:** 132–136.

Li, G., Li, B., Liu, G., and Zhang, G. (2005). Sterols from *Aspergillus ochraceus* 43. *Chin J Appl Environ Biol* **11**: 67–70

Liu, S., Dai, H., Orfali, R.S., Liu, Z., and Proksch, P. (2016) New fusaric acid derivatives from the endophytic fungus *Fusarium oxysporum* and their phytotoxicity to barley leaves. *J Agric Food Chem* **64:** 3127–3132.
